# A Lamellar Zn-Based Coordination Polymer Showing Increasing Photoluminescence upon Dehydration

**DOI:** 10.3390/molecules28155643

**Published:** 2023-07-25

**Authors:** Oier Pajuelo-Corral, Jose Angel García, Oscar Castillo, Antonio Luque, Claudio Mendicute-Fierro, Antonio Rodríguez-Diéguez, Javier Cepeda

**Affiliations:** 1 Departamento de Química Aplicada, Facultad de Química, Universidad del País Vasco/Euskal Herriko Unibertsitatea (UPV/EHU), 20018 Donostia, Spain; oier.pajuelo@ehu.eus (O.P.-C.); claudio.mendicute@ehu.eus (C.M.-F.); 2 Departamento de Física, Facultad de Ciencia y Tecnología, Universidad del País Vasco/Euskal Herriko Unibertsitatea (UPV/EHU), 48940 Leioa, Spain; joseangel.garcia@ehu.eus; 3 Departamento de Química Orgánica e Inorgánica, Facultad de Ciencia y Tecnología, Universidad del País Vasco/Euskal Herriko Unibertsitatea (UPV/EHU), 48940 Leioa, Spain; oscar.castillo@ehu.eus (O.C.); antonio.luque@ehu.eus (A.L.); 4 BCMaterials, Basque Center for Materials, Applications and Nanostructures, UPV/EHU Science Park, 48940 Leioa, Spain; 5 Departamento de Química Inorgánica, Facultad de Ciencias, Universidad de Granada, 18071 Granada, Spain; antonio5@ugr.es

**Keywords:** zinc(II), coordination polymer, lamellar structure, pyrimidine-4,6-dicarboxylate, photoluminescence properties, time-dependent density-functional theory

## Abstract

The present study reports on a 2D lamellar coordination polymer (CP) of {[Zn(µ_3_-pmdc)(H_2_O)]·H_2_O}_n_ formula (pmdc = pyrimidine-4,6-dicarboxylate). This CP is synthesized under an appropriate acid-base reaction between the gently mortared reagents in the solid state through a solvent-free procedure that avoids the presence of concomitant byproducts. The X-ray crystal structure reveals the occurrence of Zn_2_ entities connected through carboxylate groups of pmdc, which behave as triconnected nodes, giving rise to six-membered ring-based layers that are piled up through hydrogen bonding interactions. In addition to a routine physico-chemical characterization, the thermal evolution of the compound has been studied by combining thermogravimetric and thermodiffractometric data. The photoluminescence properties are characterized in the solid state and the processes governing the spectra are described using time-dependent density-functional theory (TD-DFT) with two different approaches employing different program packages. The emissive capacity of the material is further analyzed according to the dehydration and decreasing temperature of the polycrystalline sample.

## 1. Introduction

Coordination polymers (CPs) and metal–organic frameworks (MOFs), metal–organic compounds consisting of metal ions and (usually) organic ligands by means of coordination bonds, are characterized by easily tunable structures with an infinite extension in one, two or three dimensions [1,2,3,4]. The large diversity of exchangeable metal sites, a priori inexhaustible variety of organic molecules give rise to a wide range of architectures possessing specific chemical functions and physical characteristics that can be chosen by a predefined synthetic approach [5,6,7,8]. These superior features have largely attracted the interest of synthetic chemists and made CPs a fast-growing class of materials for their use in so different applications such as gas storage [9,10] and separation [11], catalysis [12], magnetism [13,14,15], electronics [16], spintronics [17,18], energy storage and conversion [19,20,21] and photoluminescence [22,23,24]. In particular, luminescent CPs are currently an important branch of these sorts of compounds and are known to provide tunable emissions derived from not only their framework but also their interplay with surrounding medium (solvents, guest molecules, composites) [25]. As a consequence, the potential application of luminescent CPs has evolved and today encompasses such different themes as the chemical sensing of ions, biomolecules, the pH of a solution, temperature and pressure [26,27,28,29]. Focusing on the inherent luminescence arising from the framework, the emission may be derived from metal ions and/or the organic ligands. In the former, the most common strategy deals with the use of lanthanide(III) ions, because their luminescence can be nurtured in the framework by capturing the energy absorbed by ligand chromophores through the well-known antenna effect when the appropriate ligands and coordination features are set [30,31]. On the other hand, organic ligand-centered emissions usually arise from *π*-electron-rich aromatic clouds or charge transfer processes between neighboring ligands, so they also play a key role in the excitation/emission scenario in addition to the aforementioned structural design. That emission capacity is often improved in CPs based on metal ions with a closed-shell electronic configuration, because these cations (i) do not cause quenching derived from d–d transitions, and (ii) diminish the vibrational motions in the framework owing to their coordination.

With particular regard to the architecture dimensionality of CPs, two-dimensional structures are being extensively investigated in an effort to better understand their underlying physics and exciting properties [32]. Within the 2D polymer-based materials, lamellar CPs consisting of tiling organic ligands linked to metal ions in a two-dimensional landscape are commonly referred to as 2D MOFs, coordination nanosheets and metal-organic layers. The weak and reversible interactions among the layers allow for the self-assembly of distinct supramolecular architectures and become from these materials into unique platforms with distinctive properties [33,34,35]. In particular, they may present optimal interaction points with external molecules; for instance, in the action as electrocatalysts, conductive materials and photoluminescent sensors, among others [36].

Bearing these ideas in mind, in the present work we have synthesized and characterized a new 2D lamellar CP by the combination of pyrimidine-4,6-dicarboxylate (pmdc) with zinc(II). The selection of pmdc is based on our previous experience with this anion which is known to establish strong chelating rings with first-row and lanthanide(III) ions [37,38,39], while it also acts as a good light absorption molecule to imbue the resulting CP with good photoluminescence properties. On its part, zinc(II) ions, with d^10^ configuration, fulfill their mission of enhancing the emissive properties of the material. The analysis of the photoluminescence is approached by a computational viewpoint in order to understand the processes governing that property. Moreover, the thermal activation of the material is also studied in order to explore the evolution of the PL owing to its dehydration.

## 2. Results and Discussion

### 2.1. Synthetic Strategy

The title compound has been synthesized by means of a less common reaction, known as a solvent-free reaction, that has been often employed for obtaining pure organic molecules rather than metal–organic compounds [40,41]. However, from 2012 onward, this alternative procedure consisting of a raw mechano-chemical synthesis followed by thermal treatment started to be employed for growing several metal-azolate frameworks, in which the resulting mixture of grinding a metal oxide or hydroxide with a diazole- or triazole-like ligand was heated in the solid state in the absence of an external solvent [42,43]. As already demonstrated, such a reaction may lead to a crystalline material of similar quality to that conventionally crystallized by the evaporation method or solvothermal procedure, with the advantage of avoiding the use of pollutant solvents, which also translates into a cheaper procedure. Apparently, the success of the oven-heated solvent-free approach lies on the appropriate selection of the reagents, because in their acid-base reaction they must promote a kind of a melt in which single crystals or at least polycrystalline powders may grow. On that basis, diazole/triazole-like ligands count on the advantage of low melting points, in such a way that, at certain temperatures, they yield melts that allow digesting metal oxides and hydroxides, which in turn lack anions that could remain in the mixture and crystallize as impurities [44]. However, the oven-heated solvent-free procedure rapidly gave some important steps and could be employed along with other sorts of ligands and metal salts that a priori could not be melted, as it is the case of ligands with a high melting point such as pmdc [39,45]. Under these circumstances, one can use hygroscopic metal salts (often hydrated salts) with a marked trend to capture water when mixed with organic anions, in such a way that the mixture, once sealed and subject to heat, may possess enough solvent as to completely mix the reagents and promote the crystallization aided by the autogenous pressure generated in the vessel. In addition to the latter, the selection of the metal salt must also take into account the possible pollution of the resulting mixture, for which anions such as nitrates and acetates that are easily decomposed under relatively low temperatures [46] have been widely recognized. Moreover, these kinds of starting salts are also easily removed from the final mixture simply by washing the sample gently with distilled water.

**Scheme 1:**
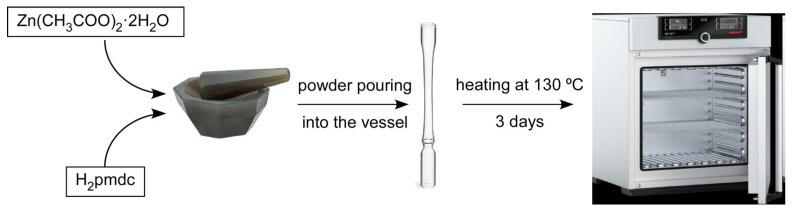
Reaction condition of the solvent-free method followed in the synthesis.

The synthesis employed for the present work is carefully designed on all the above facts and accordingly follows the reaction between jointly ground zinc(II) acetate and pyrimidine-4,6-dicarboxsylic acid. The resulting mixture is transferred to a glass vessel that, once sealed, is oven-heated at 130 °C for 3 days to favor the acid-base reaction (Scheme 1) and also enable the decomposition and evaporation of the acetic acid formed as a byproduct. In fact, the powder X-ray diffraction data acquired on the washed sample perfectly fits the pattern simulated from the single-crystal structure (Figure S3). In any case, it must also be emphasized that a solvent-free method is not always appropriate to reproduce any CPs but it may yield a crystalline compound different to the one resulting from the solvothermal procedure even if the solvent employed is water (the major solvent present in the described solvent-free procedure). In the present case, the solvothermal procedure in water leads to a previously reported 1D compound [47] that cannot be reproduced by a solvent-free procedure. However, this fact is not necessarily a disadvantage, but it allows for increasing the structural diversity of CPs.

### 2.2. Structural Description of {[Zn(µ_3_-pmdc)(H_2_O)]·H_2_O}_n_ (***1***)

**Figure 1:**
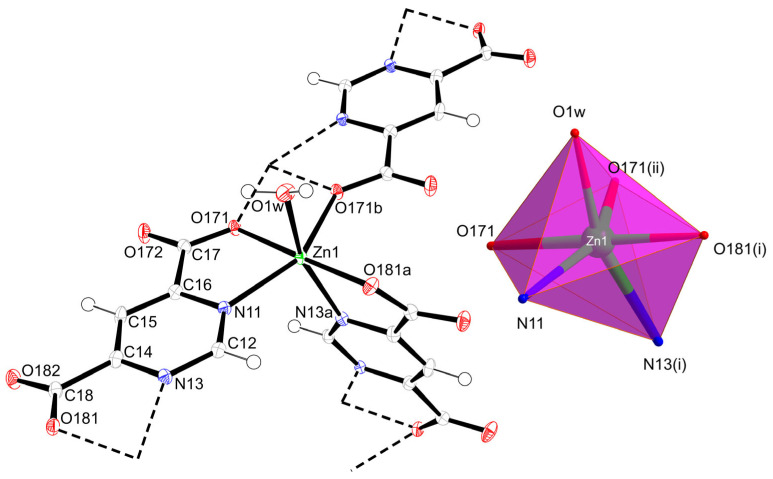
The coordination environment of compound **1** showing the ORTEP view and the polyhedron.

The synthesized CP crystallizes in the *Pbca* crystallographic space group in the form of 2D neutral layers connected to one another by means of hydrogen bonding interactions mediated by lattice water molecules. Each zinc ion is coordinated to three pmdc ligands and one coordinated water molecule in a severely distorted octahedral environment formed by the N_2_O_3_Ow donor set (Soc = 2.91, Table 1) according to the continuous shape measurements carried out with SHAPE software (version 2.1) [48] (Table S2). Two of the pmdc ligands form five-member chelate rings using one of the carboxylic oxygens and one of the nitrogen atoms of the pyrimidine ring, whereas a third pmdc ligand is coordinated in a monodentate fashion, all of which renders a µ_3_-κ^2^*N11*, *O171*:κ^2^*N13*, *O181*:κ*O171* coordination mode for pmdc ligands (Figure 1).

**Figure 2:**
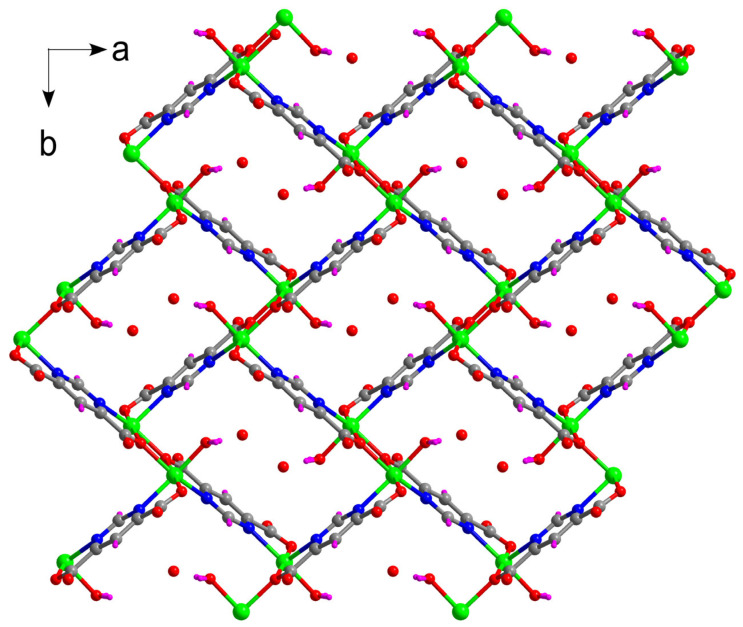
2D sheets of compound **1** showing the six-membered rectangles.

The fact that one of the pmdc ligands is coordinated perpendicularly to the other two leads to the formation of a 2D sheet by the formation of six-membered rings showing a perfect rectangle (Figure 2). The topological analysis carried out suggested that the network may be simplified as a **fes** topology, considering the aforementioned connectivity of both Zn and pmdc nodes. Furthermore, the layers are stacked along the crystallographic *c* axis by hydrogen bonding interactions between the coordination water molecules pointing to the interior of the rings, the lattice water molecules and with the O172 oxygen atom of neighboring sheets (Table S1 and Figure S1). The packing of the successive layers shows an offset among consecutive layers giving an ABAB packing. The latter prevents the presence of channels within the crystalline architecture (Figure S2).

### 2.3. Thermal Behavior

**Figure 3:**
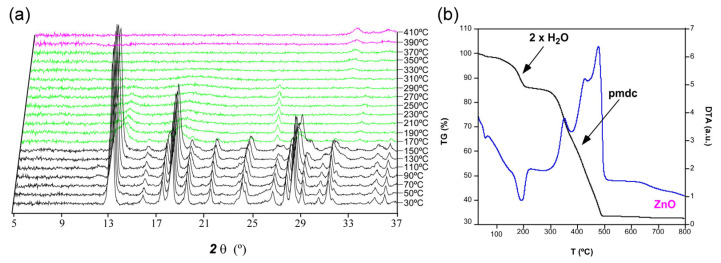
Analysis of the thermal behavior of compound **1** showing (**a**) PXRD collected in the 30–410 °C temperature range, (**b**) thermogravimetric analysis.

To study the effect of temperature on the structure of compound **1**, variable temperature powder X-ray diffractograms were collected in the 30–410 °C range in addition to a thermogravimetric analysis (Figure 3). The collected diffraction pattern at 30 °C fits well with that simulated for the structure of compound **1**. No changes are observed on the diffractograms until 170 °C, where there is a massive drop in the crystallinity of the compound that seems to be a consequence of a transformation in the architecture due to the loss of both coordinated and lattice molecules, in accordance with the mass loss observed in the TG curve that corresponds to 14.3% of the total mass. Unfortunately, given the low crystallinity of the material and the low diffraction capacity, no structural information could be extracted for the dehydrated compound (**1_an_** hereafter). This phase seems to be stable until 350 °C in agreement with the plateau observed in the TG curve, where a massive drop of the mass occurs due to the loss of the organic part to give rise to ZnO as the final residue.

In order to check the reversibility of the dehydration process, we performed a simple experiment consisting of exposing a sample of **1_an_** to a water vapor atmosphere for 5 days (see S4 section in the ESI for further details). However, the recollected sample did not revert back **1_an_** into hydrated compound **1** as confirmed by the PXRD, which showed no change with regard to the initial amorphous sample.

### 2.4. Photoluminescence Properties

**Figure 4:**
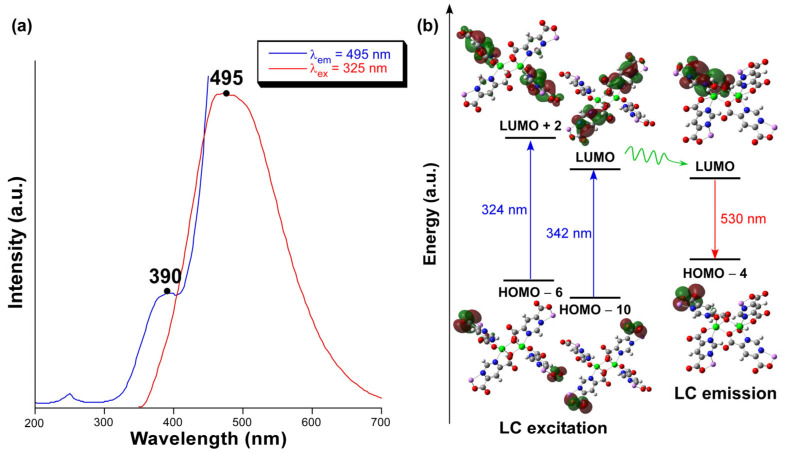
(**a**) Experimental excitation (blue) and emission (red) spectra of **1** at RT. (**b**) Schematic representation of the most relevant calculated excitation and emission lines.

The photoluminescent properties of compound **1** have been explored given the fact that the closed-shell nature on Zn(II) could enhance ligand-centered emissions. Accordingly, the excitation and emission spectra are recorded at room temperature using a polycrystalline sample. The emission spectrum (Figure 4a) covers the 360–700 nm spectral range with the appreciation of a wide band peaking at ca. 495 nm that slowly decays up to 700 nm. No significant differences are observed in the emission spectrum upon excitation at 375 nm excitation source (Figure S8). On its part, the excitation spectrum recorded at an emission wavelength of 495 nm is formed by a wide band with the maximum centered at 390 nm which is followed by a strong band that cannot be completely observed due to its overlap with the emission maximum. The bands observed in the excitation and emission spectra can be assigned to π-π* electronic transitions of the pmdc ligand as observed in other CPs built from a pmdc ligand [38,49]. In fact, their shape is quite similar to the spectra recorded for the free H_2_pmdc ligand (where λ_ex,max_ = 335 and λ_em,max_ = 435 nm), although a clear red shift of ca. 60 nm may be a claim for both the excitation and emission maxima of compound **1** with respect to the ligand (Figure S10). The emission quantum yield (QY), recorded with an integrating sphere, was estimated to be of 1.5 (2)% for the polycrystalline sample.

Computational calculations have been conducted using the TDDFT methodology as implemented in Gaussian in order to provide further insights into the photoluminescence mechanism occurring in this compound. As observed in Figure 4b, the excitation of the compound (based on a dimeric model described in Figure S12) may be described by two main excitation lines (at 342 and 324 nm), each of which is ascribed to two electronic transitions: (i) the lower energy line (λ_ex_ = 342 nm) corresponds to LUMO ← HOMO-10 and LUMO + 1 ← HOMO-11 transitions while (ii) the most energetic line is best ascribed to LUMO + 2 ← HOMO-6 and LUMO + 3 ← HOMO-7 transitions. An additional significant excitation is also found at 296 nm (see Table S3), which corresponds to the S_9_ excited state and is assigned to two main transitions with low weights. As observed in Figure S14, that wavelength fills within the absorption band shown in the diffuse reflectance spectrum recorded on the polycrystalline sample of **1**. Therefore, considering the notable difference between the absorption and excitation bands (∆ ≈ 140 nm, with no overlap between the main bands), that high-energy excited state, in case it is populated, is expected to transfer the charge to the lowest-energy S_1_ in order to proceed with the luminescent emission. Despite the variety of molecular orbitals involved in these transitions, a detailed examination reveals that the four HOMOs and LUMOs are closely related since they consist of π orbitals centered on carboxylate groups and π* orbitals extended over the pyridine and carboxylate groups of the ligand (see Figure S13). This fact, in addition to the proximity of the transitions, which pertain to two overlapping bands, seems to indicate that all electronic transitions can be assigned to the same excited state and they are a result of a vibronic progression. The geometry optimization of the first excited state of the model leads to a subtle breakage of the planarity among initially (in the ground state geometry) coplanar pmdc ligands. The analysis of the transitions involved in the emission on the later geometry shows that main line peaks at 530 nm and is assigned to LUMO → HOMO-4 which a similar intraligand charge transfer (ILCT) to the one described for the excitation process.

**Figure 5:**
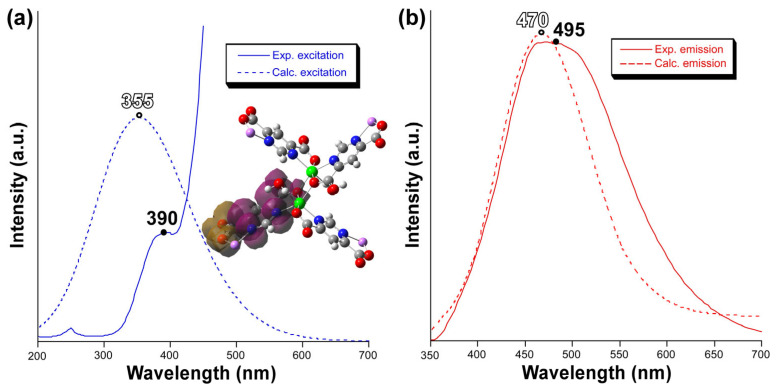
Experimental (solid line) and calculated (dotted line) spectra for the (**a**) excitation and (**b**) emission processes. The inset shows the plot of the ED difference over the ground state geometry. Note also that the main wavelengths at the band maxima are also shown.

On another level, the excitation and emission spectra were also computed by the ESD module implemented in the ORCA program. Following this strategy, the calculated excitation spectrum fairly reproduced the experimental one by showing a main band centered at 355 nm, which is blue-shifted 35 nm with regard to the main band observed in the experimental measurement (Figure 5). This result is in agreement with the previous main excitations calculated with Gaussian shown in Figure 4. In fact, a plot showing the electron density difference between the S_0_ and S_1_ states over the model at S_0_ geometry confirms the ILCT character of the excitation process. As observed in the plot, the negative electron density (golden surface) located on the π cloud of the carboxylate group of one of the pmdc ligands and the positive density at the π cloud of the aromatic ring confirms the aforementioned described carboxylate → aromatic ring electronic transition. On its part, regarding the emission, the calculated spectrum gives a very good correspondence with the experimental one by showing a slightly blue-shifted (25 nm) band compared to the experimental signal, with the maximum centered at 470 nm. The calculated fluorescence at the optimized geometry for the S_1_ state confirms, therefore, the Kasha-like nature of the compound given that the lowest-lying singlet is the main excited state involved in the light-emission process.

With the aim of decreasing the non-radiative vibrational quenching, measurements were repeated at a temperature of 15 K without any noticeable modification in the excitation nor in the emission spectra, apart from the expected increase in intensity (Figure S9b). The main emission band emission lifetime was also monitored with the aim of studying the long-lasting emission capacity of the compound. Accordingly, decay curves were recorded at the emission maxima (495 nm) and fitted to the following multiexponential expression: (I_t_ = A_0_ + A_1_exp(t/*τ*_1_) + A_2_exp(t/*τ*_2_)). The estimated lifetime for the fitting at room temperature is 4.25 ns, which is no longer at 13 K with a calculated lifetime of 5.00 ns (Figure S10). Recorded emission lifetimes indicate the fluorescence nature of the emission derived from the short lifetimes obtained.

**Figure 6:**
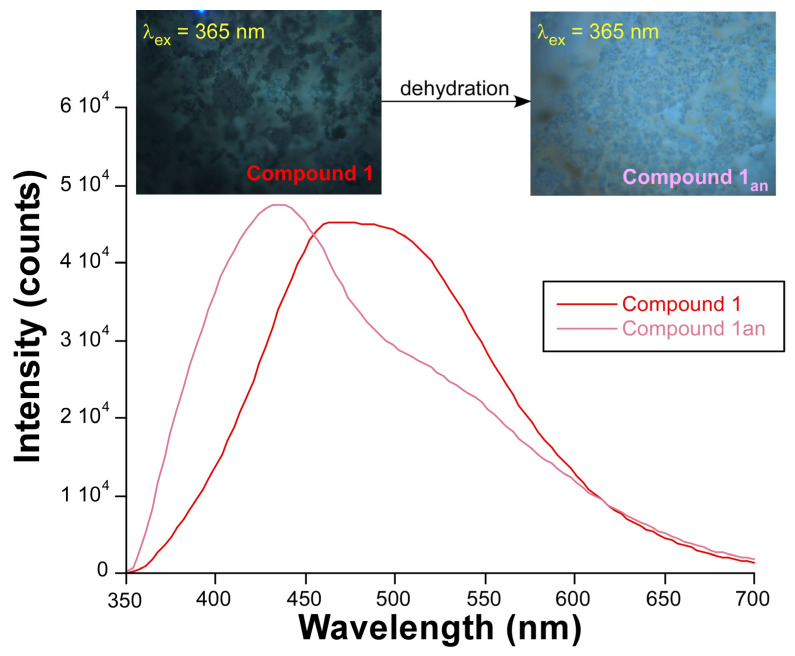
Room temperature emission spectra of compounds **1** and **1_an_** showing the micrographs taken over polycrystalline samples illuminated with UV light.

At last, taking advantage of the detailed study conducted on the thermal behavior of the compound, we decided to further explore the photoluminescence properties of the fully dehydrated material to see if relevant changes could occur with the loss of both lattice and coordinated water molecules. This attempt is motivated by the fact that water molecules in the inner coordination sphere are known to significantly quench the luminescence of CPs due to the coupling of the donor–acceptor energy gaps with the O–H oscillators, in such a way that an increase of the photoluminescence may be expected [50,51]. Accordingly, a polycrystalline sample of **1** is heated in an oven at 250 °C (at the plateau of the TG curve shown above) for 4 h and then left to cool down to room temperature. Under laser monochromatic excitation light (λ_ex_ = 325 nm), the completely anhydrous sample (**1_an_**) shows a slightly different emission profile consisting of a main band sited at 435 nm and an intense shoulder (with half of the emission of the main band) peaking at ca. 550 nm. Therefore, irrespective of the low-energy shoulder present in **1_an_**, the dehydration produces a blue shift in the emission of about 60 nm, which slightly moves the emitted color to a purer blue as it can be corroborated by both the chromaticity diagrams referred to the CIE1931 code (see Figure S13) and the micrographs taken on the microscope over both crystalline samples (Figure 6). The excitation spectrum recorded on **1_an_** focusing the main emission line shows no significant change in the bands although the main band around λ_ex_ = 400 nm (observed for the hydrated compound **1**) is not fully shown due to its proximity to the emission band. Taking into account that the emission intensity in the steady-state spectra cannot be employed to quantitatively estimate the emission capacity, the QY was measured for **1_an_** using the same experimental setup and it was shown to be almost doubled compared to the hydrated compound (2.7(7)% for **1_an_** vs. 1.5(2)% for **1**).

The sample of compound **1_an_** has also been studied at a low temperature for comparative purposes with the pristine material. When the temperature is dropped from RT to 15 K, the emission band is slightly red-shifted (the λ_em_ for the maximum is shifted from 435 to 445 nm) and the high-energy region of the band presents a certain structure, which may be related with a more defined vibrational structure to the excited and ground states of the compound. Moreover, the emission lifetime is much smaller for **1_an_** compared to **1** and can only be reliably estimated at a low temperature (1.5 ns, see Figure S15).

## 3. Materials and Methods

### 3.1. Synthesis of {[Zn(µ_3_-pmdc)(H_2_O)]·H_2_O}_n_ (***1***)

A solvent-free procedure was followed for the synthesis of compound **1**. It consisted of hand-grinding a mixture of H_2_pmdc (0.0306 g, 0.15 mmol) and Zn(CH_3_COO)_2_·2H_2_O (0.036 g, 0.15 mmol) and placing the homogeneous mixture, which was sealed into a glass tube and slowly heated up to 130 °C for 48 h in an autoclave and then slowly cooled down to room temperature for the synthesis of polycrystalline samples. The resulting product was gently washed with distilled water several times in order to remove the acetic acid formed as a byproduct of the acid-base reaction. Yield: 95–100% (based on metal). Anal. Calcd. for C_6_H_6_N_2_O_6_Zn (%): C, 26.94; H, 2.26; N, 10.47; Zn, 24.44. Found: C, 26.78; H, 2.46; N, 10.35; Zn, 24.32.

Colorless single crystals were obtained by performing the same procedure, but keeping the setpoint temperature at 110 °C for a longer period (96 h) and slowly cooling down to room temperature at a 2 °C/h rate. However, it is worth noticing that crystals of **1** are obtained in low yield (45–50%) as part of a mixture with low crystalline zinc(II) acetate, making this reaction procedure less recommended.

### 3.2. Physical Measurements

Elemental analyses (C, H, N) were carried out on a Euro EA Elemental Analyser. The content of zinc in the samples was estimated by means of ICP-AES measurements with a Horiba Yobin Yvon Activa spectrometer. An IR spectrum was collected on an FTIR 8400S Shimadzu spectrometer in the 4000–400 cm^−1^ spectral region using KBr pellets. Thermal analyses (TG/DTA) were performed on a TA Instruments SDT 2960 thermal analyzer under a synthetic air atmosphere (79% N_2_, 21% O_2_) with a heating rate of 5 °C/min.

### 3.3. X-ray Diffraction Data Collection and Structure Determination

Single crystal diffraction data collection was carried out at 100(2) K on an Agilent Technologies Supernova diffractometer (λ_Cu-κα_ = 1.5418 Å). Data reduction was performed with the CrysAlisPro program [52]. The crystal structure was solved by direct methods using SHELXT program [53] and refined by full-matrix least-squares on F^2^ including all reflections employing the WINGX crystallographic package. Hydrogen atoms were introduced in the difference Fourier map as fixed contributions using riding models with isotropic thermal displacement parameters of 1.2 times those of their parent atoms. The most relevant refinement data can be found in Table 2. CCDC 2271672 contains the supplementary crystallographic data for this paper. These data can be obtained free of charge via http://www.ccdc.cam.ac.uk/conts/retrieving.html (accessed on 22 June 2023) (or from the CCDC, 12 Union Road, Cambridge CB2 1EZ, UK; Fax: +44-1223-336033; E-mail: deposit@ccdc.cam.ac.uk).

X-ray diffraction patterns were collected on a Philips X’PERT powder diffractometer with Cu-Kα radiation (λ = 1.5418 Å) in the 5 < 2θ < 50° range with a step size of 0.02° and an acquisition time of 2.5 s per step at 25 °C. Indexation of diffraction patterns was performed using the FULLPROF crystallographic package (pattern matching analysis) [54] based on the cell parameters and space group obtained from single crystal X-ray diffraction measurements. Variable temperature powder X-ray diffraction was performed under an ambient atmosphere with a heating range of 5 °C/min and acquiring a diffractogram in the 5 < 2θ < 38° range every 20 °C in the 30–410 °C range.

### 3.4. Photoluminescence Measurements

Photoluminescence spectra were recorded on an Edinburgh Instruments FLS920 spectrometer conducted using a close cycle helium cryostat. All measurements were performed under a high vacuum (of ca. 10^−9^ mbar) to avoid the presence of oxygen or water in the sample holder. For steady-state measurements, an IK3552R-G HeCd continuous laser (325 nm) and a Müller-Elektronik-Optik SVX1450 Xe lamp were employed as the excitation source. On the other hand, the decay curves were measured using a pulsed laser diode LDH-P-C-375 (λ = 375 nm) to estimate the lifetime on the ligand-centered emission. The analysis of the fluorescence was detected with a photomultiplier tube (PMT) coupled to the spectrometer. The overall quantum yield (%) was measured in solid state and at room temperature by means of a Horiba Quanta–φ integrating sphere using an Oriel Instruments MS257 lamp as the excitation source and an iHR550 spectrometer from Horiba to analyze the emission. Photographs taken on irradiated polycrystalline samples of compound **1** were performed with an Olympus optical microscope illuminated with a Hg lamp and equipped with a CCD detector.

### 3.5. Computational Calculations

Computational calculations were carried out on a suitable model of compound **1** that consists of a dimeric excerpt of [Zn_2_(pmdc)_4_Li_4_(H_2_O)_2_] in which the four outer chelating moieties of the pmdc originally occupied by Zn(II) ions have been replaced by Li(I) ions in order to simulate the periodicity of the structure and also balance the charges of the framework. The initial X-ray coordinates were optimized with the ORCA 5.0.3 program [55,56]. These calculations were performed at the DFT level of theory with the Becke three parameter hybrid functional with the non-local correlation functional of Lee–Yang–Parr (B3LYP) [57,58] and employing a ZORA Hamiltonian in order to account for relativistic effects [59] as well as the atom-pairwise dispersion correction with the Becke–Johnson damping scheme (D3BJ) [60,61], a ZORA-def2-TZVP basis set for C, H, N, and O atoms, and a SARC-ZORA-QZVPP basis set for Zn, including coulomb and exchange contributions [62,63]. Some restraints were used to avoid a complete rearrangement of the fragment and a frequency calculation was also conducted to confirm that an energy minimum was achieved. PL spectra were calculated with two different strategies. On the one hand, TD-DFT as implemented in the Gaussian 16 package [64] was employed with B3LYP functional along with a 6-31G+(d) basis set [65] for all atoms but for the central zinc cation, for which the LANL2DZ [66] basis set along with the corresponding effective core potential (ECP) was used. The 40 lowest excitation states were calculated by the TD-DFT method. Results were analyzed with the GaussSum program package [67] and molecular orbitals plotted using GaussView 6 [68]. On the other hand, the computed excitation and emission spectra were also obtained with the ORCA 5.0.3 program with the TDDFT methodology and modules implemented to perform calculations based on the optimized geometries of the S_0_ and S_1_ states and the spin–orbit coupling (SOC) matrix elements.

## 4. Conclusions

A layered 2D metal–organic material, characterized as {[Zn(µ_3_-pmdc)(H_2_O)]·H_2_O}_n_ (**1**), has been obtained by the reaction of the pyrimidine-4,6-dicarboxylic acid with zinc acetate under solvent-free conditions at a high temperature. The synthetic procedure yields a crystalline product grown in the melt formed during the acid-base reaction in which water is afforded from the reagents and the acetic acid formed as a byproduct is evaporated from the vessel. The triconnected pmdc linker establishes 2D sheets based on Zn_2_ entities that show six-membered rings, in such a way that an overall **fes** topological framework is built. The compound presents photoluminescence properties under UV irradiation based on π-π* electronic transitions centered at the pmdc ligand in good agreement with the TDDFT calculations performed, which imbue the solid with a dark blue emission characterized by a low emissive yield (QY of 1.5%). The thermodiffractometric data reveal that the compound presents a unique loss of mass corresponding to the release of both lattice and coordinated water molecules at room temperature–230 °C temperature range. In spite of the fact that the crystal structure loses its order during the transformation, the dehydration promotes a non-innocent change in the luminescence of the material since the anhydrous product, of [Zn(µ_3_-pmdc)]_n_ (**1_an_**) formula, displays a comparatively stronger light-blue emission that almost doubles the QY (up to 2.7%) of the pristine crystalline compound. Lowering the temperature of both samples provokes an increase of their emissive capacity mainly due to the decrease of the non-radiative quenching (probably due to the freezing of molecular vibrations) because no significant changes are observed in the observed lifetimes recorded at room and low temperatures.

## Figures and Tables

**Table 1:** Coordination distances for compound **1** (Å) ^1^.

Zn1-N11	2.179(3)	Zn1-O171(ii)	2.125(3)
Zn1-N13(i)	2.223(3)	Zn1-O181(i)	2.021(3)
Zn1-O171	2.092(2)	Zn1-O1w	2.089(3)

^1^ Symmetry codes: (i) x + 1/2, −y + 1/2, −z; (ii) −x + 2, −y + 1, −z.

**Table 2:** Single crystal X-ray diffraction data and structure refinement details of compound **1**.

Compound	1
Empirical formula	C_6_H_6_N_2_O_6_Zn
Formula weight (g mol^−1^)	267.51
Crystal system	Orthorhombic
Space group	*Pbca*
a (Å)	9.8493(2)
b (Å)	12.2231(2)
c (Å)	13.6321(3)
V (Å^3^)	1641.15(6)
Reflections collected	9760
Unique data/parameters	1660/137
Rint	0.0712
GoF (S) ^a^	1.136
R_1_^b^/wR^2^ [I > 2σ(I)] ^c^	0.0418/0.0954
R_1_^b^/wR^2^ [all] ^c^	0.0508/0.0994

^a^ S = [∑w(F_0_^2^ − F_c_^2^)^2^/(N_obs_ − N_param_)]^1/2^. ^b^ R_1_ = ∑||F_0_| − |F_c_||/∑|F_0_|; ^c^ wR^2^ = [∑w(F_0_^2^ − F_c_^2^)^2^/∑wF_0_^2^]^1/2^; w = 1/[σ^2^(F_0_^2^) + (aP)^2^ + bP] where P = (max(F_0_^2^,0) + 2Fc^2^)/3 with a = 0.0328 and b = 3.6167.

## Data Availability

The data supporting this study’s findings are available from the corresponding author upon reasonable request.

## Supplementary Materials

The following supporting information can be downloaded at: https://www.mdpi.com/article/10.3390/molecules28155643/s1, Figure S1. Representation of the C ligand bonds.; Figure S2. Packing of compound **1** along the (a) a axis and (b) c axis viewing directions; Figure S3. Pattern-matching analysis of polycrystalline sample of compound **1**; Figure S4. FTIR spectrum of compound **1**; Figure S5. Comparison of the FTIR spectra of compound **1** and the free H_2_pmdc ligand. Figure S6. Excitation spectrum of compound 1 taken at room temperature at λem = 495 nm; Figure S7. Emission spectrum of compound **1** measured at room temperature at λem = 375 nm; Figure S8. Excitation (λem = 495 nm) (a) and emission (λem = 325 nm) (b) spectra of compound 1 recorded at a temperature of 13 K; Figure S9. Excitation (λem = 495 nm) (a) and emission (λem = 325 nm) (b) spectra of compound **1** recorded at a temperature of 13 K; Figure S10. Decay curves of compound **1** recorded at (a) room temperature and (b) 13 K at an emission wavelength of 495 nm; Figure S11. Dimeric model employed for the calculations performed with Gaussian and ORCA programs; Figure S12. MOs of the dimeric model of compound **1** involved in the main excitation and emission; Figure S13. Diffuse reflectance spectrum recorded on polycrystalline sample of compound **1** at room temperature; Figure S14. Chromaticity diagrams showing the integrated emission pattern for compound **1** (left) and **1_an_** (right) at room temperature; Figure S15. (a) Comparative excitation spectra of compound **1_an_** collected over the main emission line. (b) Emission spectrum of **1_an_** recorded at 15 K; Figure S16. Decay curves of compound **1_an_** recorded at room temperature at an emission wavelength of 445 nm; Figure S17. Decay curves of compound **1_an_** recorded at room temperature at an emission wavelength of 445 nm. Table S1. Hydrogen bonding interactions (Å, °) of compound **1**; Table S2. Continuous Shape Measurements for the coordination environment for compound **1**. The lowest SHAPE values are shown in bold blue, indicating best fits; Table S3. Calculated main excitation and emission energies (nm), singlet electronic transitions and associated oscillator strengths of model 1 in gas phase.

## Sample Availability

Samples of the compound reported in this work is available from the authors.
