# Supplementary material for: A Lamellar Zn-Based Coordination Polymer Showing Increasing Photoluminescence upon Dehydration
Source: Molecules. 2023 Jul 25;28(15):5643. doi: 10.3390/molecules28155643 (PMC10419880; doi:10.3390/molecules28155643)
Supplement: Supplementary file 1 [file molecules-28-05643-s001.zip › molecules-2493679-supplementary.pdf]

Electronic Supplementary Information for:

A Lamellar Zn-Based Coordination Polymer Showing Increasing Photoluminescence upon Dehydration

Oier Pajuelo-Corral [1], Jose Angel García [2], Oscar Castillo [3,4], Antonio Luque [3,4], Claudio Mendicute-Fierro [1], Antonio Rodríguez-Diéguez [5] and Javier Cepeda [1,*]

[1] Departamento de Química Aplicada, Facultad de Química, Universidad del País Vasco/Euskal Herriko Unibertsitatea (UPV/EHU), 20018 Donostia, Spain; oier.pajuelo@ehu.eus (O.P.-C.); claudio.mendicute@ehu.eus (C.M.-F.)
[2] Departamento de Física, Facultad de Ciencia y Tecnología, Universidad del País Vasco/Euskal Herriko Unibertsitatea (UPV/EHU), 48940 Leioa, Spain; joseangel.garcia@ehu.eus
[3] Departamento de Química Orgánica e Inorgánica, Facultad de Ciencia y Tecnología, Universidad del País Vasco/Euskal Herriko Unibertsitatea (UPV/EHU), 48940 Leioa, Spain; oscar.castillo@ehu.eus (O.C.); antonio.luque@ehu.eus (A.L.)
[4] BCMaterials, Basque Center for Materials, Applications and Nanostructures, UPV/EHU Science Park, 48940 Leioa, Spain
[5] Departamento de Química Inorgánica, Facultad de Ciencias, Universidad de Granada, 18071 Granada, Spain; antonio5@ugr.es
* Correspondence: javier.cepeda@ehu.es; Tel.: +34-943015409

Contents:

S1. Structural details.

S2. Continuous Shape Measurements (CShMs).

S3. Powder X-ray Diffraction Analysis.

S4. Study of the dehydration reversibility of compound $\mathbf{1_{an}}$.

S5. FT-IR spectroscopy.

S6. Photoluminescence measurements and calculations.

S1. Additional structural details.

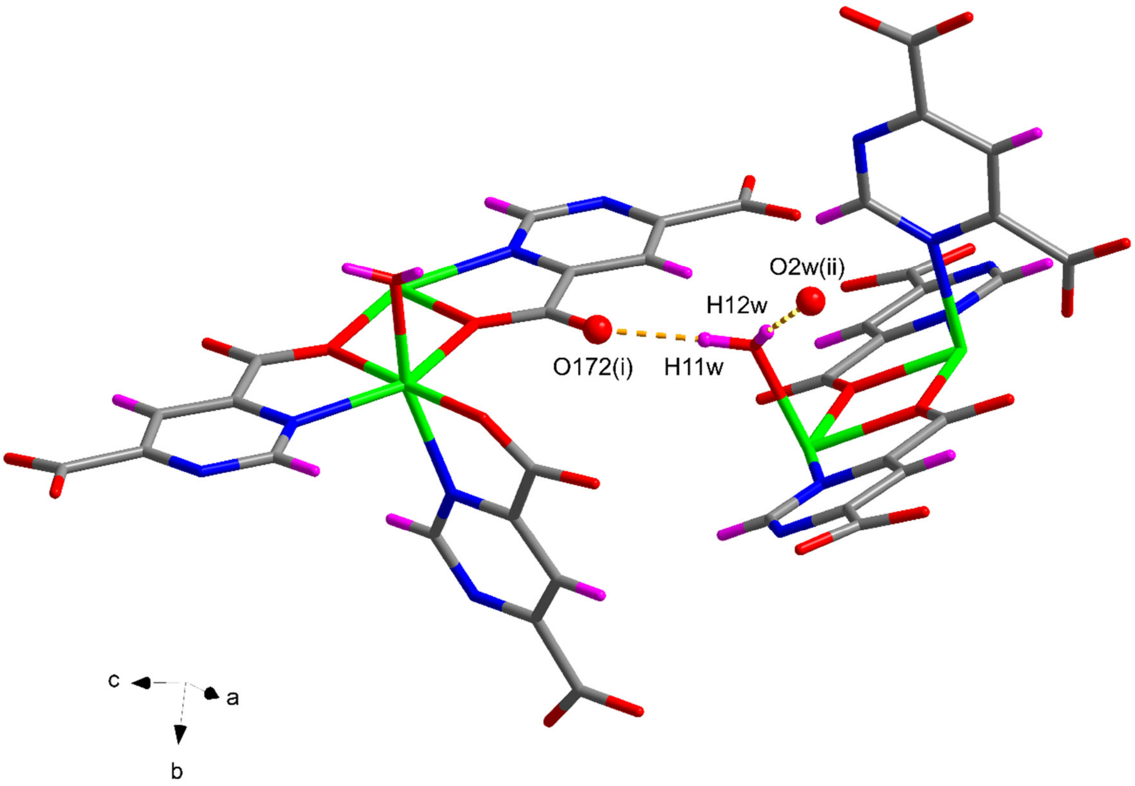

Figure S1. Representation of the C ligand bonds.

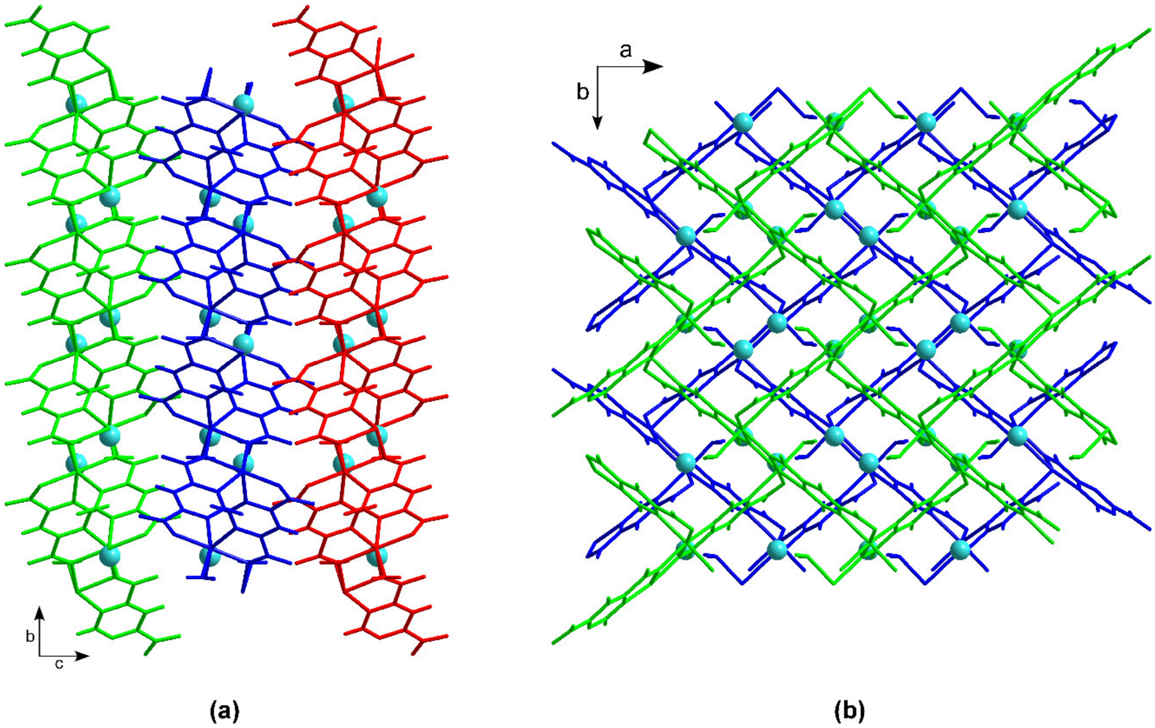

Figure S2. Packing of compound 1 along the (a) *a* axis and (b) *c* axis viewing directions.

Table S1. Hydrogen bonding interactions (Å, °) of compound **1**.[a]

D–H···A[b]	*D–H*	*H···A*	*D···A*	*D–H···A*
O1w–H11w···O172(i)	0.86	1.87	2.725(4)	177.5
O1w–H12w···O2w(ii)	0.86	1.84	2.693(5)	169.3

[a] Symmetry codes: (i) –x + 3/2, –y + 1, z + 1/2; (ii) –x + 1, –y + 1, –z. [b] D: donor. A: acceptor.

S2. Continuous Shape Measurements (CShMs).

Table S2. Continuous Shape Measurements for the coordination environment for compounds **1**. The lowest SHAPE values is shown in bold blue, indicating best fits.

Codes:

HP-6 1 D6h Hexagon

PPY-6 2 C5v Pentagonal pyramid

OC-6 3 Oh Octahedron

TPR-6 4 D3h Trigonal prism

JPPY-6 5 C5v Johnson pentagonal pyramid J2

**Structure [ML6]**	**HP-6**	**PPY-6**	**OC-6**	**TPR-6**	**JPPY-6**
**Comp 1-Zn1**	32.557	21.916	**2.907**	11.294	25.502

S3. Powder X-ray Diffraction Analysis.

As observed in Figure S3, the diffraction pattern acquired on the polycrystalline sample obtained by solvent-free procedure fits perfectly the unit cell parameters (and calculated Bragg positions) of the single-crystal data, which confirms the purity of the sample.

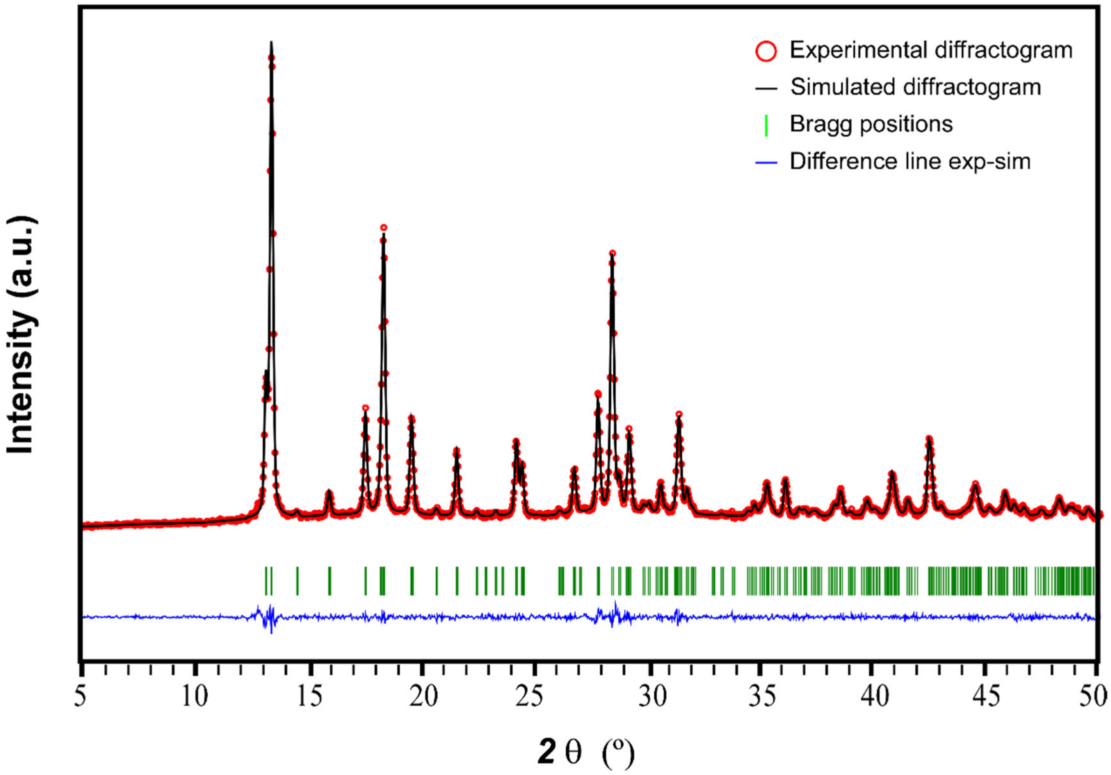

Figure S3. Pattern-matching analysis of polycrystalline sample of compound **1**.

S4. Study of the dehydration reversibility of compound $\mathbf{1}_{an}$.

With the aim of checking if the completely anhydrous compound $\mathbf{1}_{an}$ can revert back to compound **1** by means of a water adsorption process, we carried out a simple experiment in which the sample of $\mathbf{1}_{an}$ was exposed to water vapor. For the experiment, we placed the sample in a small glass vial and, in turn, we placed the vial into a bigger vessel containing distilled water. The vials were left at in a sand-bath at 50 ºC for 5 days, in such a way that water vapor was gently generated. Finally, the vials were open, the sample recovered and analyzed by PXRD technique, by which a similar pattern to the initial amorphous compound was obtained.

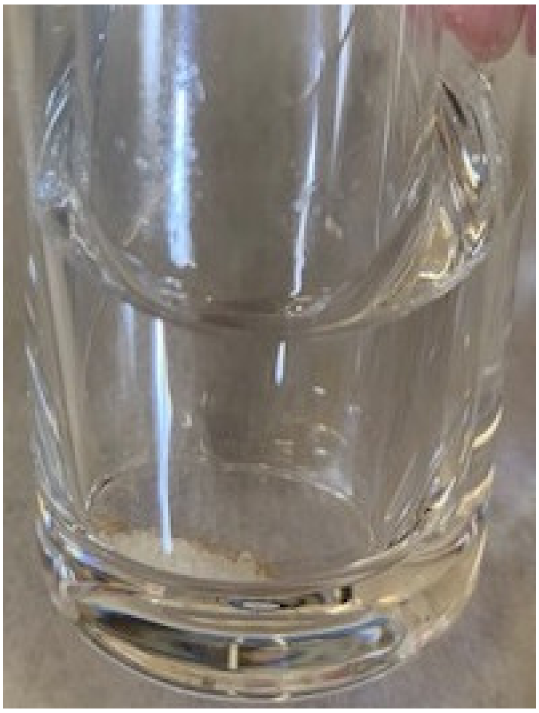

Figure S4. Photograph showing the exposure of sample of compound $\mathbf{1}_{an}$ to water vapours. The sample is contained in a vial which, in turn, is placed inside a bigger vial containing water.

S5. FT-IR spectroscopy.

Infrared spectroscopy was used as an initial characterization technique to check the presence of pmdc in compound **1** (Figure S4). The spectrum shows a wide band in the 3550-3100 cm^{-1} which can be assigned to the characteristic band of OH vibrations of water molecules and to the C-H vibration of the pyrimidinic ring of pmdc. The intense peaks that appear at approximately 1650 and 1360 cm^{-1} correspond to the asymmetric and symmetric stretching vibration of the carboxylate groups. In the 1300-1000cm^{-1} region several medium- and weak-intensity peaks appear that can be attributed to the distortions originated in the aromatic ring of pmdc ligand. The bands appearing below 550 cm^{-1} are originated from the M-O and M-N bonds. A comparison of the FTIR spectrum with that of the free ligand can be found in Figure S5.

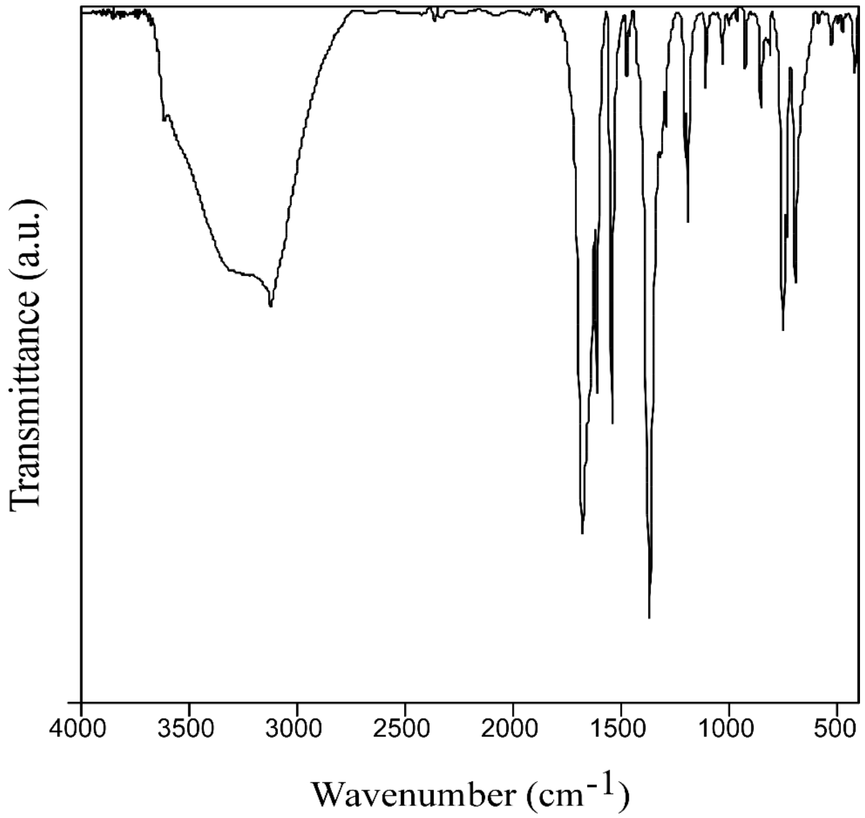

Figure S5. FTIR spectrum of compound **1**.

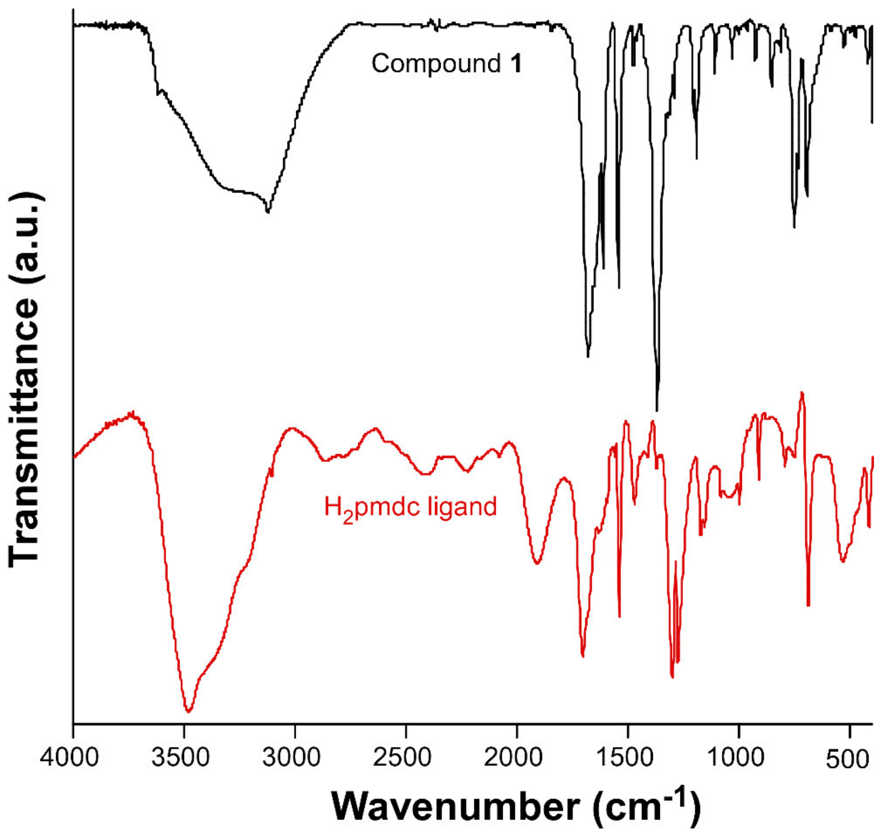

Figure S6. Comparison of the FTIR spectra of compound **1** and the free H_2pmdc ligand.

S6. Photoluminescence measurements and calculations.

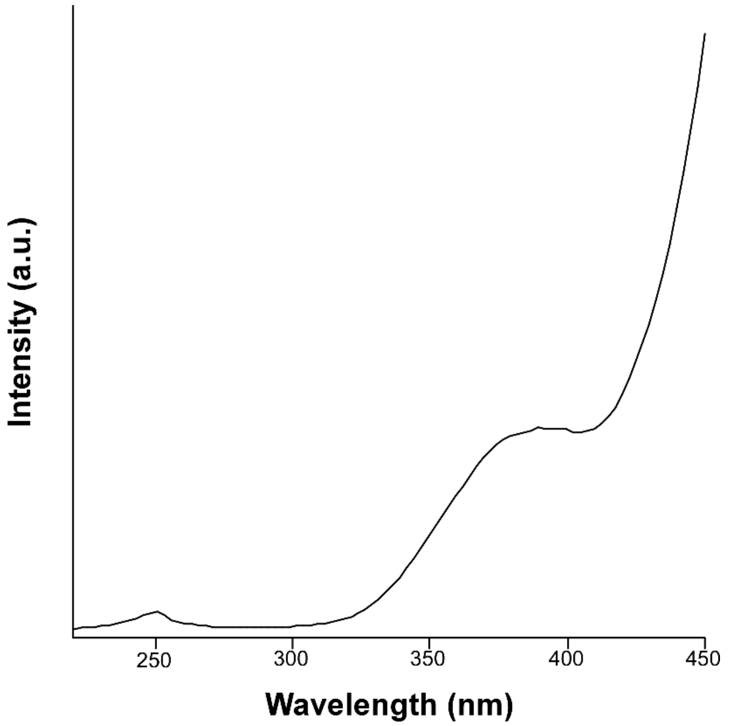

Figure S7. Excitation spectrum of compound **1** taken at room temperature at λ_{em} = 495 nm.

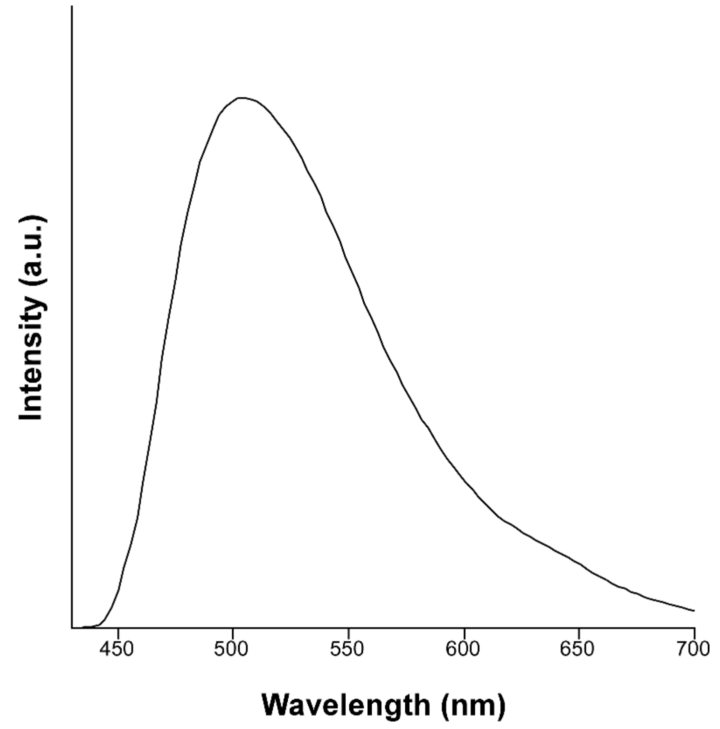

Figure S8. Emission spectrum of compound **1** measured at room temperature at λ_{em} = 375 nm.

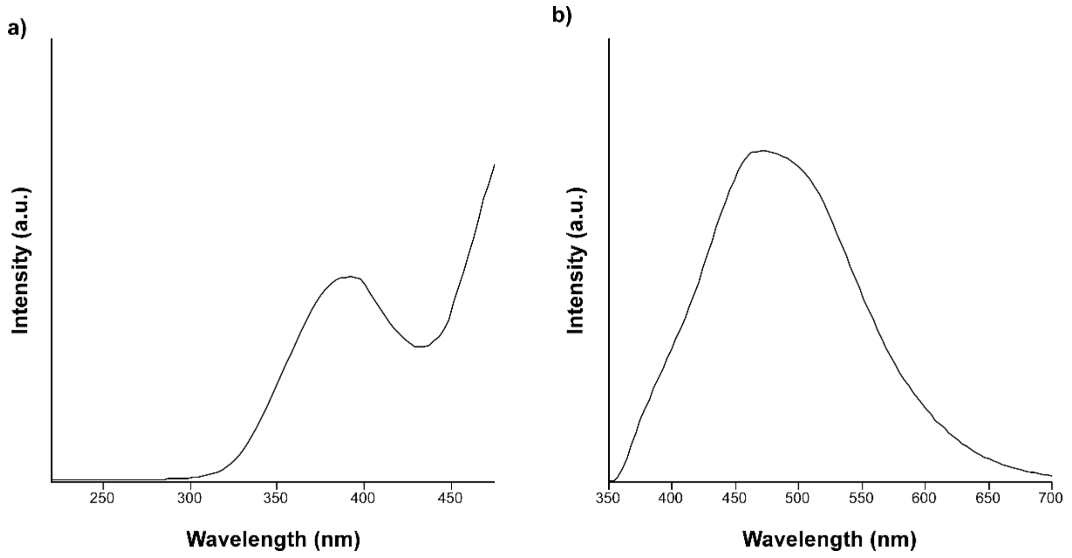

Figure S9. Excitation (λ_{em} = 495 nm) (a) and emission (λ_{em} = 325 nm) (b) spectra of compound **1** recorded at a temperature of 13 K.

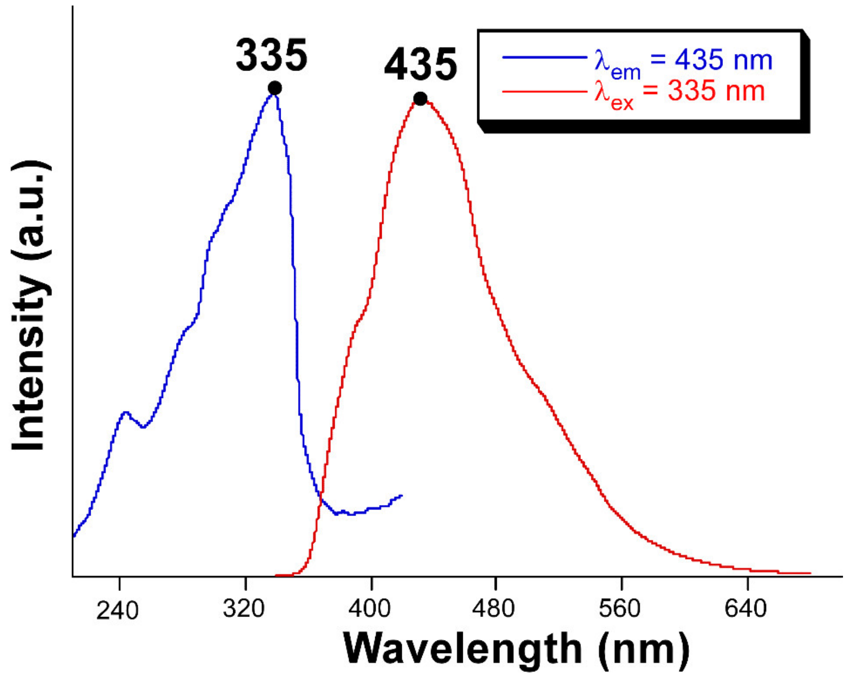

Figure S10. Excitation (λ_{em} = 495 nm) (a) and emission (λ_{em} = 325 nm) (b) spectra of compound **1** recorded at a temperature of 13 K.

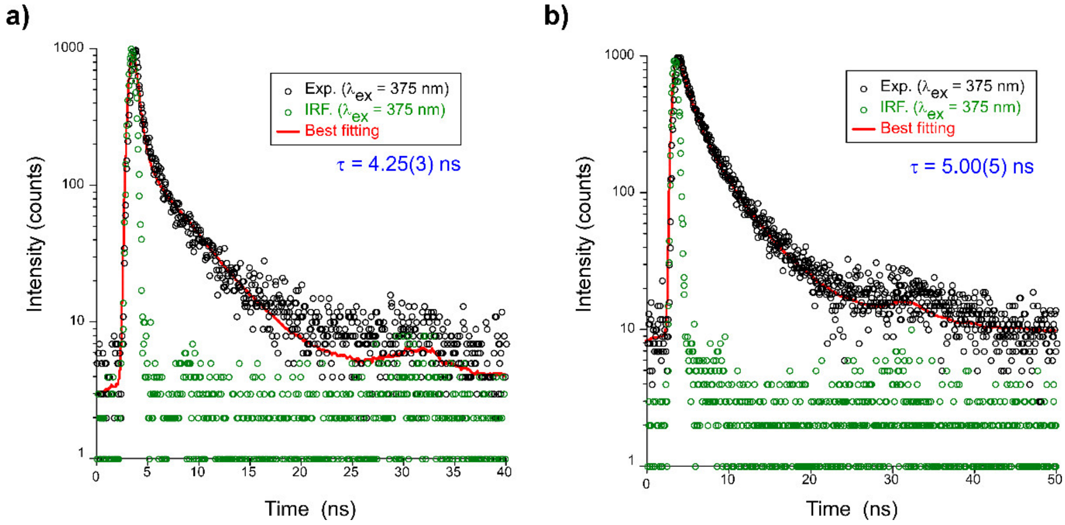

Figure S11. Decay curves of compound **1** recorded at a) room temperature and b) 13 K at an emission wavelength of 495 nm.

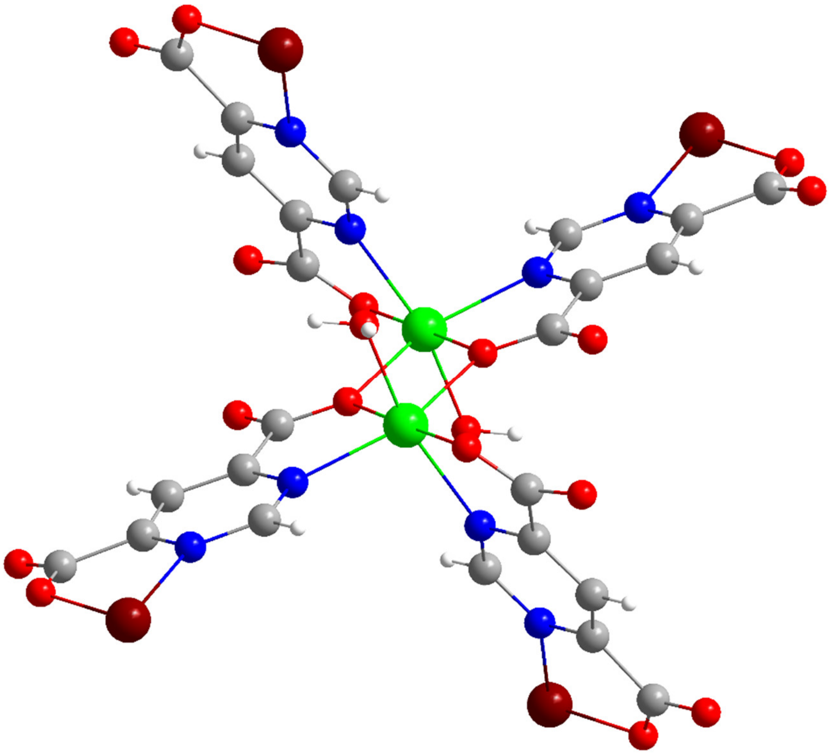

Figure S12. Dimeric model employed for the calculations performed with Gaussian and ORCA programs.

Excitation

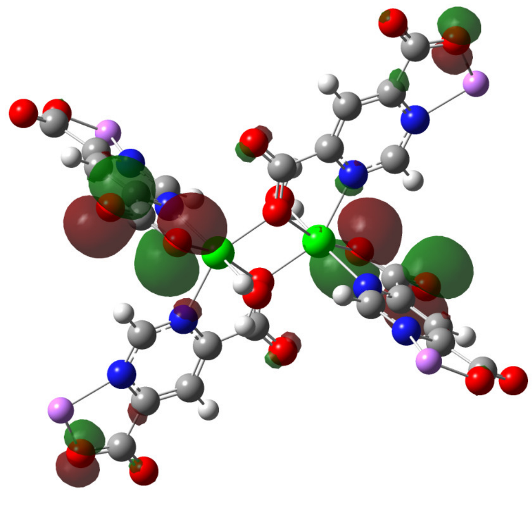

HOMO–15

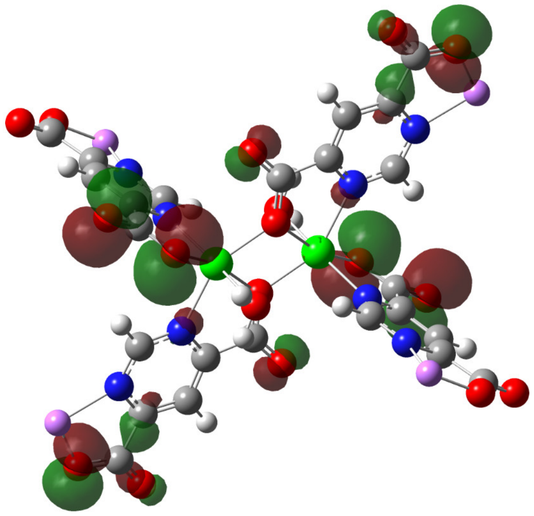

HOMO–14

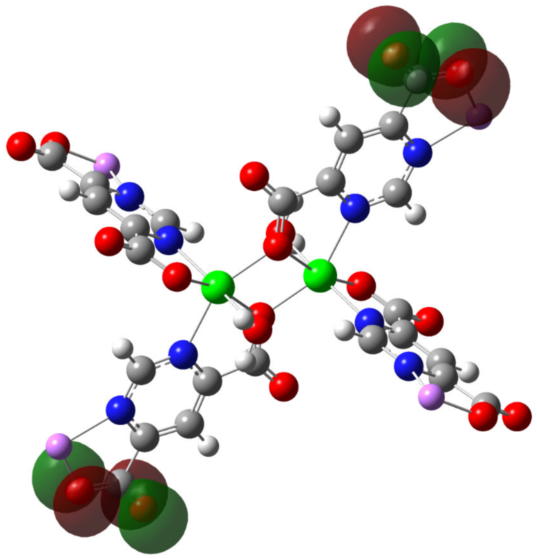

HOMO–11

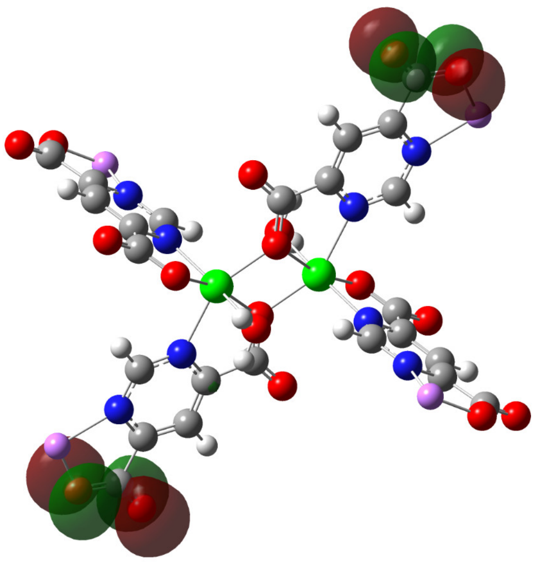

HOMO–10

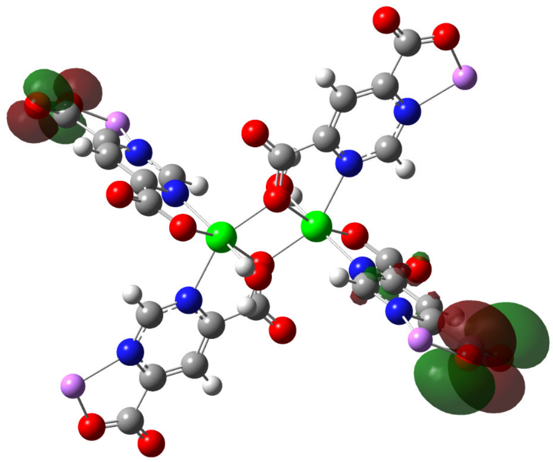

HOMO–7

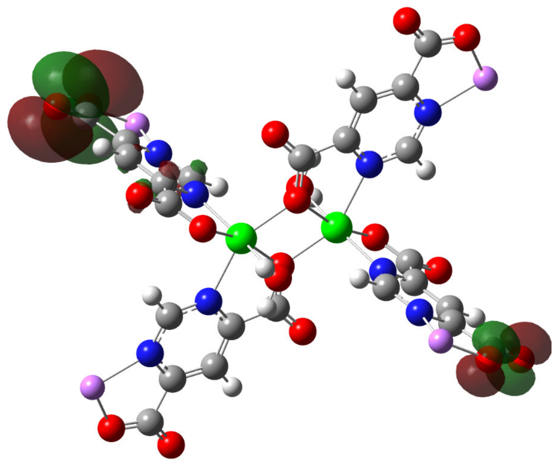

HOMO–6

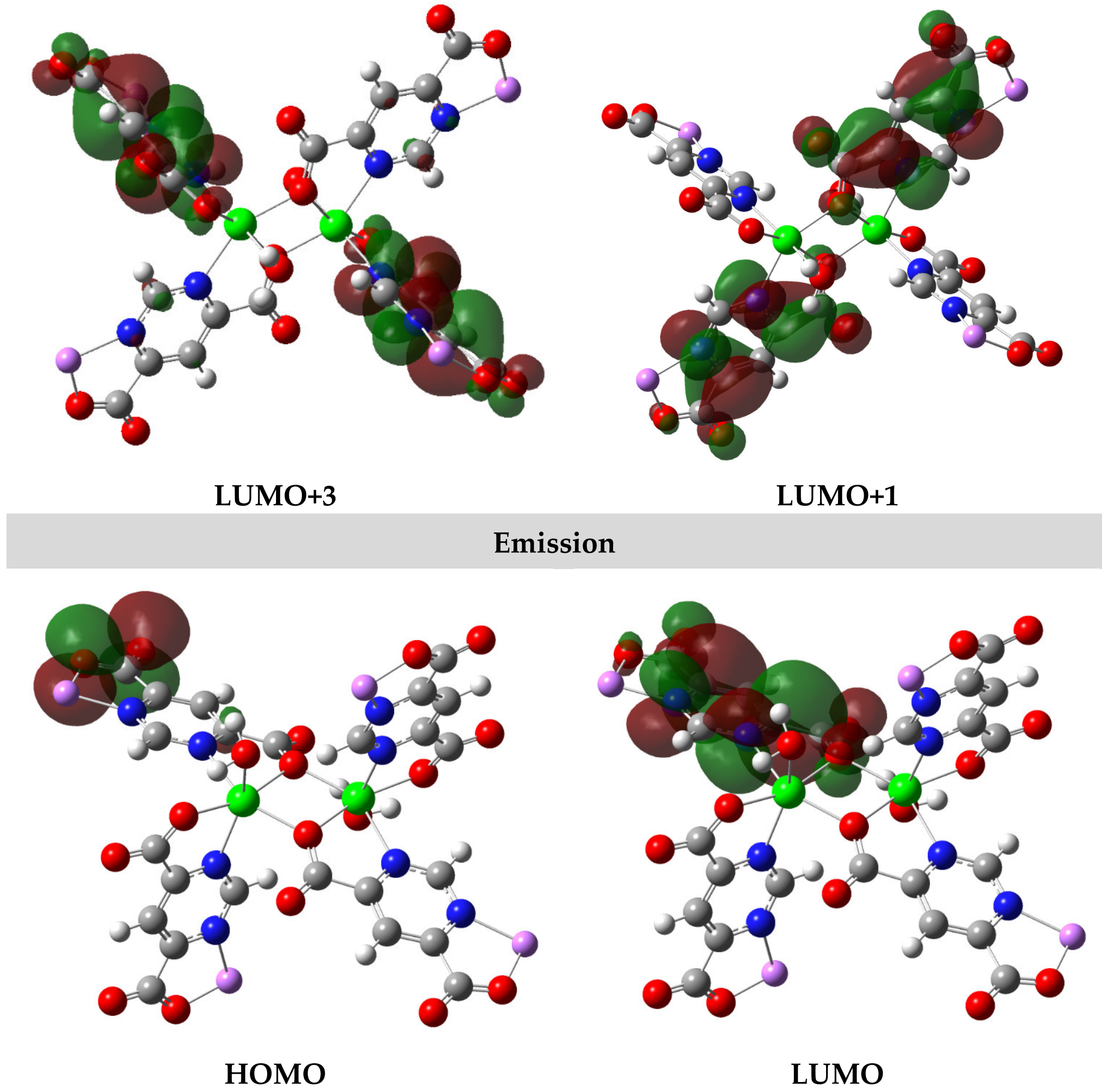

Figure S13. MOs of the dimeric model of compound **1** involved in the main excitation and emission.

Table S3. Calculated main excitation and emission energies (nm), singlet electronic transitions and associated oscillator strengths of model 1 in gas phase.

Exp. λ (nm)	Calcd. λ (nm)	Significant contributions	Osc. strength (a.u.)
		Excitation energies	
390	296	HOMO – 15 → LUMO + 3 (42%)<br>HOMO – 14 → LUMO + 2 (45%)	0.0192
390	342	HOMO – 10 → LUMO (78%)	0.0321
390	324	HOMO – 6 → LUMO + 2 (75%)	0.0393
		Emission energies	
495	530	HOMO – 4← LUMO (99%)	0.0282

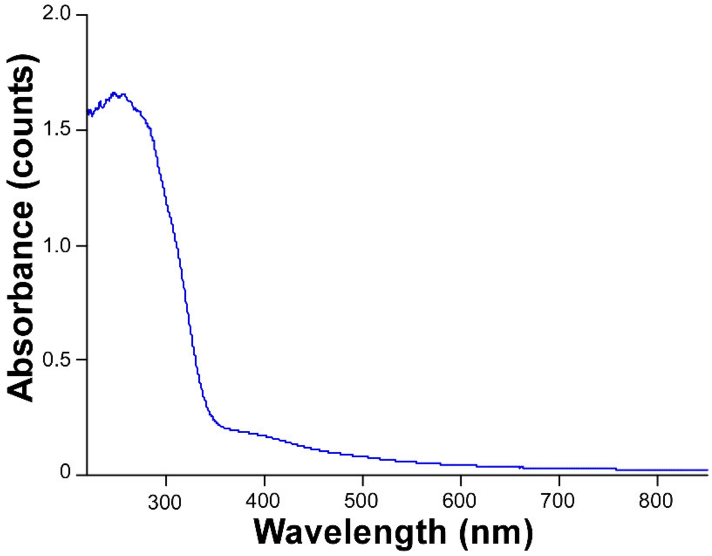

Figure S14. Diffuse reflectance spectrum recorded on polycrystalline sample of compound **1** at room temperature.

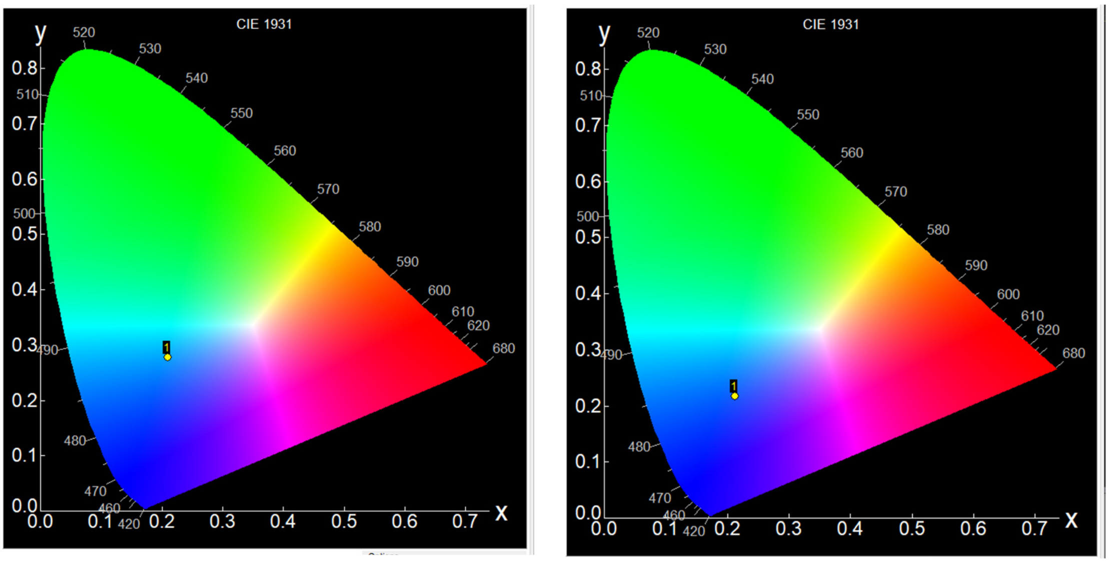

Figure S15. Chromaticity diagrams showing the integrated emission pattern for compound **1** (left) and $\mathbf{1}_{an}$ (right) at room temperature.

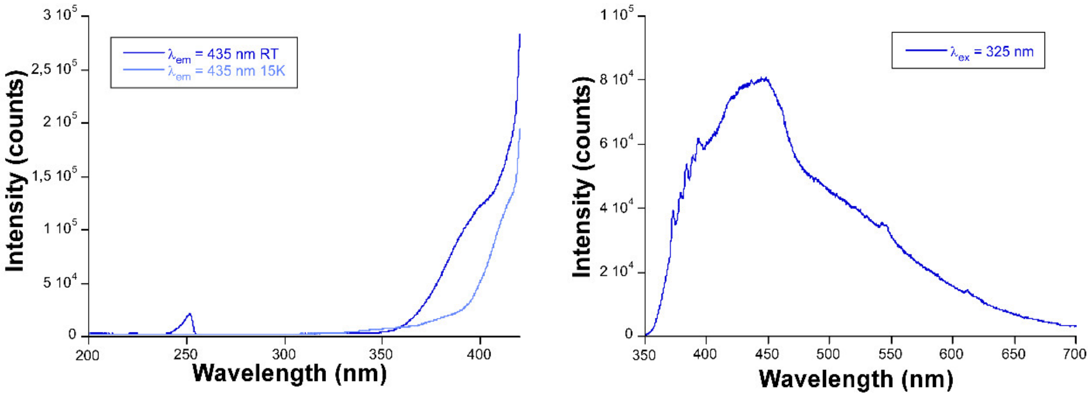

(a) **(b)**

Figure S16. **(a)** Comparative excitation spectra of compound **1an** collected over the main emission line. **(b)** Emission spectrum of **1an** recorded at 15 K.

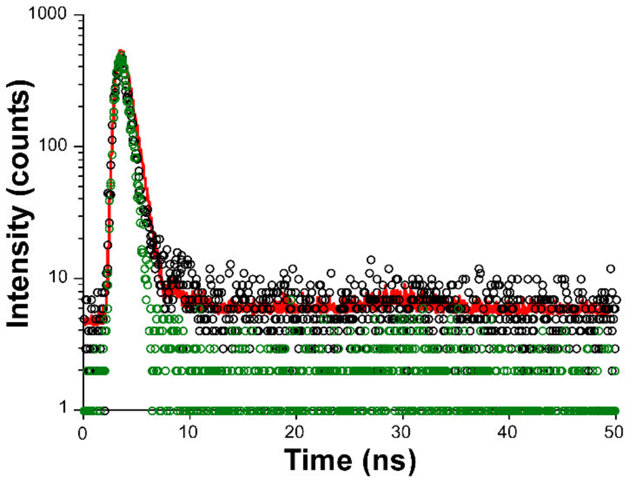

Figure S17. Decay curves of compound **1an** recorded at room temperature at an emission wavelength of 445 nm.
